# Aesthetic Rehabilitation Medicine: Enhancing Wellbeing beyond Functional Recovery

**DOI:** 10.3390/medicina60040603

**Published:** 2024-04-05

**Authors:** Lorenzo Lippi, Martina Ferrillo, Luigi Losco, Arianna Folli, Marco Marcasciano, Claudio Curci, Stefano Moalli, Antonio Ammendolia, Alessandro de Sire, Marco Invernizzi

**Affiliations:** 1 Department of Health Sciences, University of Eastern Piedmont “A. Avogadro”, 28100 Novara, Italy; lorenzolippi.mt@gmail.com (L.L.); arianna.folli23@gmail.com (A.F.); stefano.moalli@libero.it (S.M.); marco.invernizzi@med.uniupo.it (M.I.); 2 Translational Medicine, Dipartimento Attività Integrate Ricerca e Innovazione (DAIRI), Azienda Ospedaliera SS. Antonio e Biagio e Cesare Arrigo, 15121 Alessandria, Italy; 3 Department of Health Sciences, School of Dentistry, University of Catanzaro “Magna Graecia”, 88100 Catanzaro, Italy; martina.ferrillo@unicz.it; 4 Plastic Surgery Unit, Department of Medicine, Surgery and Dentistry, University of Salerno, Via Salvador Allende, 43, 84081 Baronissi, Italy; llosco@unisa.it; 5 Plastic Surgery, Experimental and Clinical Medicine Department, Division of Plastic and Reconstructive Surgery, “Magna Graecia” University of Catanzaro, 88100 Catanzaro, Italy; m.marcasciano@unicz.it; 6 Physical Medicine and Rehabilitation Unit, Department of Neurosciences, ASST Carlo Poma, 46100 Mantova, Italy; 7 Physical and Rehabilitative Medicine, Department of Medical and Surgical Sciences, University of Catanzaro “Magna Graecia”, 88100 Catanzaro, Italy; ammendolia@unicz.it; 8 Research Center on Musculoskeletal Health, MusculoSkeletalHealth@UMG, University of Catanzaro “Magna Graecia”, 88100 Catanzaro, Italy

**Keywords:** aesthetic rehabilitation, disability, botulinum toxin, platelet-rich plasma, hyaluronic acid, shock wave therapy, rehabilitation

## Abstract

Although rehabilitation medicine emphasizes a holistic health approach, there is still a large gap of knowledge about potential interventions aimed at improving overall wellbeing through cosmetic interventions. Therefore, this narrative review investigates the role of different rehabilitative techniques in enhancing aesthetics, quality of life, and psychosocial wellbeing for patients with disabilities. The study follows the SANRA framework quality criteria for a narrative review. Literature searches across PubMed/Medline, Web of Science, and Scopus identified articles focusing on rehabilitation strategies within the aesthetic rehabilitation domain. The review identified evidence supporting injection procedures, such as Botulinum Toxin, Platelet-Rich Plasma, Hyaluronic Acid, Ozone, and Carboxytherapy, and assessing their applications in several disabling disorders. Additionally, physical therapies like Extracorporeal Shock Wave Therapy, Laser Therapy, Microcurrent Therapy, Tecar Therapy, and physical exercises were explored for their impact on cutaneous microcirculation, cellulite treatment, wound healing, and scar appearance improvement. Lastly, the manuscript underlines the role of manual therapy techniques in addressing both physical discomfort and aesthetic concerns, discussing their effectiveness in adipose tissue therapy, scar tissue mobilization, and regional fat thickness reduction. Taken together, this review emphasizes the role of a multidisciplinary approach, aiming to provide valuable insights into potential benefits for both functional and aesthetic outcomes.

## 1. Introduction

Rehabilitation medicine aims to bring the patient closer to an extended concept of health, intended as physical and mental wellbeing, based on the principles of the International Classification of Functioning, Disability and Health (ICF) [1], with the objective of enhancing physical function, increasing social participation, and contributing to a full recovery of the patient’s social life [2].

Consequently, in rehabilitation interventions the specialist also finds himself having to consider the aesthetic aspect of diseases and disabilities, as this is an integral part of the patient’s state of health. Indeed, aesthetic treatments might enhance psychological wellbeing and social perception [3], increasing Quality of Life (QoL) in several detrimental conditions [4,5,6].

A classic example is oncological rehabilitation, where, in addition to reconstructive medicine, aesthetic intervention in breast cancer patients [7] might reduce psychological distress, and cosmetic rehabilitation after maxillofacial cancer in affected subjects might increase QoL [8]. These interventions are recommended in the literature, which considers aesthetic intervention on a par with medical treatment in cancer patients and as closely related to their QoL [9,10]. Moreover, aesthetics in rehabilitation is also crucial in the consideration and prescription of limb prostheses, burn rehabilitation, and management of scars, which might also have strong physical, functional, and emotional repercussions [11,12].

Indeed, in modern medicine, the scientific literature underpins the importance of aesthetics in the private and public life of patients, which should be preserved in health and sickness. These aspects should be implemented in healthcare environments and especially in rehabilitation facilities [13,14]. Accordingly, since physical and psychological wellbeing are closely linked, and recovery of the state of good health cannot ignore the recovery of a socially accepted aesthetic appearance, a branch of rehabilitation medicine is arising that might specifically treat the aesthetic appearance of patients with disabilities, “Aesthetic Rehabilitation Medicine”.

The physiatrist has many weapons at his disposal in order to enhance aesthetics of patients while treating disabilities. Physical therapies, such as Laser Therapy, Tecar Therapy, Electrotherapy, and Extracorporeal Shock Wave Therapy (ESWT), might be utilized for an aesthetic purpose and are described in the literature as effective, safe, and well tolerated by patients in the treatment of localized fat [15]. Interventional therapies with botulinum toxin, growth factors, or hyaluronic acid, as well as carboxytherapy and ozone therapy, can also be used for both aesthetic and rehabilitative purposes in the treatment of localized fat, cellulite, rejuvenation, dermal tissue repair, acne, and skin dyschromia [16]. In addition, the effects of physical exercise and manual therapy should not be underestimated, having aesthetic and curative effects on dysmorphic self-perception and mental wellbeing [17]. Furthermore, localized facial exercises have hinted at positive outcomes in facial rejuvenation and might reduce wrinkles, although more evidence should be gathered for the indications to be conclusive [18].

Therefore, the aim of this comprehensive review is to systematically assess the role of different rehabilitative techniques and interventions routinely utilized in a rehabilitation setting, which are capable of improving aesthetics, quality of life, and psychosocial wellbeing.

## 2. Materials and Methods

This narrative review adhered to the SANRA framework quality criteria [19]. Literature searches were conducted across multiple databases, including PubMed/Medline, Web of Science (WoS), and Scopus. Controlled keywords connected to aesthetic rehabilitation were employed, including “Aesthetic Rehabilitation”, “Physical Therapies”, “Botulinum Toxin”, “Laser Therapy”, “Tecar Therapy”, and “ESWL”. The search strategy, constructed using the SPIDER tool presented in Table 1, focused on physical therapies, quality of life, and functional restoration within aesthetic rehabilitation.

Between September 2023 and January 2024, two independent reviewers (L.L. and A.F.) conducted a comprehensive literature search across multiple databases. The identified studies underwent an eligibility screening process conducted by two reviewers. When consensus was not possible, a third reviewer (A.d.S.) was asked to reach the final decision. The inclusion criteria were established to address the primary research question: “What are the potential rehabilitation strategies in aesthetic rehabilitation?”

Eligible articles focused on rehabilitation strategies in the aesthetic rehabilitation field, including different physical therapies routinely used in a rehabilitation setting and with potential cosmetic effects. The studies assessed the rehabilitation impact on improving functional outcomes, quality of life, and psychosocial wellbeing among individuals undergoing aesthetic rehabilitation.

Exclusion criteria were studies in languages other than English, full text being unavailable, and conference abstracts or theses.

Data extraction and synthesis were performed using a qualitative method. Two reviewers (L.L. and A.F.) independently extracted and synthesized data from eligible studies. In case of disagreement, a third reviewer (A.d.S.) was asked to reach a consensus. Due to the heterogeneity of the included studies and the narrative review design, a qualitative approach to synthesis was utilized, presenting all outcome data in a narrative way.

## 3. Multicomponent Role and Synergies in Aesthetic Rehabilitation

A multimodal and multidisciplinary approach is relevant in the whole field of medicine. Reconstruction following trauma, surgical oncology, or any detrimental condition (i.e., lymphoedema, sequelae of massive weight loss) is gradually becoming more closely related to patients’ aesthetic concerns, with the aim of lessening psychological discomfort and improving patient satisfaction and QoL [20,21,22,23]. The pursuit of aesthetic rehabilitation matches this novel approach perfectly.

The scientific trend of a tailor-made and aesthetic-“friendly” reconstructive surgery that is far more aware of and attentive to donor site morbidity and patient discomfort, along with patient desires and expectations, has been ongoing for the last two decades. Breast reconstruction was the forerunner branch within plastic reconstructive surgery; post-bariatric surgery and reconstruction of the head and neck region followed the path, along with growing attention to the impact of post-oncological reconstruction on elderly patients [24,25,26,27].

Breast reconstruction provides the opportunity to correct deformities and lessen psychological discomfort related to the disease and the radical operation; therefore, it can enhance patients’ QoL [28,29,30]. Over the years, various techniques to improve aesthetic and functional results in breast reconstruction have been proposed. Prosthetic breast reconstruction was shifted from a traditional two-stage operation to a single-stage procedure [31,32]. Single-stage subcutaneous/prepectoral breast reconstruction reduces postoperative pain and discomfort caused by the dissection of the pectoralis major muscle while maintaining a good cosmetic outcome without any dynamic distortion of the implant. Patients can rapidly recover arm mobilization and can be discharged in a timely manner with a quicker return to everyday activities [32,33].

In recent years, post-bariatric reconstructive surgery has evolved significantly. It was advanced both by the increasing incidence of obesity and by the growing popularity of bariatric weight loss surgeries. On the one hand, patients experience a gradual improvement in obesity-related comorbidities; on the other hand, rapid weight reduction brings a subsequent increase in skin folds. Consequently, most of the patients report their clothes no longer fit, social relationships are impacted, or hygiene issues; aesthetic concerns might, among others, be related to the abdomen, mons pubis, hip region, and gluteal region [34]. Post-bariatric surgery after massive weight loss is not a way out at no cost; the overall complication rates can vary up to 78% [34,35,36,37]. Nevertheless, these patients decide to undergo great tissue resection, and will also accept scar visibility for the functional and contour improvements resulting from greater resection [38].

Body contouring procedures in massive weight loss patients were associated with an increase in QoL and high patient satisfaction [39,40]. Patients’ aesthetic satisfaction is sometimes reported as higher than that for the surgery because it also takes into account a functional improvement. Improved walking ability, well-fitting clothes, and the relief of skin problems are not visible, but they are non-negligible features of an increased self-confidence [38].

In recent years, the ageing population has become a prominent focus within the field of reconstructive surgery [41]. Elderly patients, often characterized by complex medical histories, age-related changes in tissue quality, and longer rehabilitation time present a distinct set of challenges that demand specialized care [42,43]. The main cornerstones of a successful approach to this patient population include careful patient/procedure selection aimed at reducing complication rate and the reduction of reconstructive stages, with a single-stage approach preferred to reduce the overall stress on a fragile cohort [44,45,46,47].

Therefore, for elderly patients, adopting the proper reconstructive technique is paramount. It should be tailored to the effects that surgery has on patient satisfaction and QoL. Crucial considerations in the elderly population should include minimizing surgical invasiveness, employing a one-stage procedure, facilitating early discharge, expediting recovery, and ensuring a timely return to routine activities through the implementation of a well-structured rehabilitation programme. These factors highlight the need for a precise and patient-centred approach in the surgical decision-making process for elderly individuals undergoing reconstructive procedures.

## 4. Injection Procedures in Aesthetic Rehabilitation

### 4.1. Botulinum Toxin Injections

Botulinum toxins (BTXs) are extracted from Clostridium botulinum, a Gram-positive, spore-forming bacterium, and can be serologically differentiated into eight types (i.e., A, B, C1, C2, D, E, F, and G) [48].

Despite the antigenic variations among them, these toxins have similar functions and can be administered as intramuscular and intradermal injections. When an intramuscular injection is performed, the toxin causes the proteolysis of a synaptosomal-associated protein of 25 kDa (SNAP-25) that is essential for acetylcholine release at the neuromuscular junction, resulting in a reduced postsynaptic muscle contraction that lasts about 3 months [48,49].

BTX type A (BTX-A) is commonly used in cosmetic medicine as injections in the frontal–periocular and frontal–glabellar regions to smooth out expression roughness [50]. Indeed, it can modulate the contraction of the mimic muscles promoting skin relaxation and reducing the depth of the expression lines producing a lifting effect [51].

BTX is also used to treat several head and neck disorders, especially when the conditions are primarily of muscular origin (e.g., blepharospasm, strabismus, and torticollis) [52].

In recent years, intervention on the masticatory muscles with BTX-A has been shown to reduce the activity of muscles in patients affected by masseter hypertrophy, bruxism, and muscular-related temporomandibular disorders [53,54]. Indeed, these myofascial orofacial pain conditions are associated with increased muscle tension and pain secondary to excessive masticatory muscle activity that lead to difficulty in performing daily activities (e.g., chewing, talking, swallowing, and yawning) with a significant reduction of health-related QoL (HRQoL) [55].

A reduction of pain symptoms with an improvement in the range of motion would be expected after reducing muscle hyperactivity with BTX [53]. In this context, several randomized controlled trials (RCTs) showed the efficacy of BTX as injections into the masseter muscles with doses of BTX-A that ranged from 25 U to 300 U, and they reported a significant improvement in both range of motion and pain reduction in orofacial disorders [56,57,58,59].

An interesting RCT was conducted by De Carli et al. [60] to compare the use of low-level GaAlAs laser and BTX (30 U) in the treatment of myofascial pain, and it showed that both therapies were effective in reducing pain. Furthermore, in 2022, Delcanho et al. [53] conducted a systematic review on this topic and concluded that scientific evidence was available to support the use of BTX injections for treatment of masseter hypertrophy and for myogenous TMDs, and interestingly, the authors reported no adverse side effects in any of the studies.

BTX is also used for the treatment of involuntary repetitive or twisting spasms of masticatory muscles in patients affected by oromandibular dystonia [61]. This rare neurological condition significantly affects QoL, considering its effects on chewing, swallowing, and talking, with consequent social embarrassment and cosmetic defacement. The condition is characterized by absence of spasms during sleep and daytime spasms that are aggravated by stress and lead to persistent grinding [62].

Treatment with BTX has been widely investigated in this context. Muscles and doses of BTX are usually individually determined for each patient in relation to symptoms and occlusal forces, and the injection are performed until the patients are satisfied with the effect [63].

Sinclair et al. [62] conducted a longitudinal study of the effects of BTX injections. Based on the involved muscle, they administered 25 U for the masseter, 15 U for the temporalis, 7.5 U for the external pterygoid, 5 U for the anterior digastric, and 7.5 U for the platysma. The median number of treatments per patient was five, with no correlation among the clinical form of dystonia, the total number of injections, and the time between injections (*p*-value > 0.05). In conclusion, the authors showed that BTX treatment was effective for all clinical forms with minimal morbidity [62].

BTX is also approved for symptomatic treatment of hemifacial spasm and blepharospasm, and a review by Jost et al. [64] analysed data from 55 controlled studies and showed a success rate for BTX of approximately 90%. Moreover, BTX was showed to be effective also in patients affected by Meige syndrome, a condition in which oromandibular dystonia is accompanied by blepharospasm, and the recommended site dose fell between 1.25 U and 2.5 U per site, up to a maximum of 5 U per site [65,66].

On the other hand, animal studies suggest that weakening the masseters with BTX could lead to accelerated mandibular osteopenia [67,68]. A study found that a BTX injection into the masseter muscle of adult mice induced significant molecular changes in bone and muscle as early as 2 days post-intervention, leading to microanatomical alterations in both tissues by day 14. These changes included increased mRNA levels of bone resorption markers and muscle atrophy-related genes, as well as reductions in masseter muscle mass and fibre diameter, accompanied by subchondral bone loss in the mandibular heads [67]. At the same time, a second study conducted on adult rabbits revealed that despite minimal disruption to mastication, BTX-induced paralysis of the masseter resulted in notable and persistent bone loss, particularly at the temporomandibular joint. This bone loss is attributed to underloading of the mandibular condyle and molar area, as evidenced by decreased bone quantity and quality, even after 12 weeks [68].

In conclusion, BTX injections have been shown to be a viable therapeutic solution for different orofacial conditions, especially in patients affected by central pain syndromes [69] whose symptoms did not improve after conventional treatments, although the potential skeletal consequences of this intervention need to be considered [67,68].

### 4.2. Platelet-Rich Plasma (PRP) Therapy

In recent decades, platelet-rich plasma (PRP) has been widely applied in the fields of dentistry, orthopaedic surgery, rehabilitation, and plastic surgery for the treatments of alveolar bone defects, musculoskeletal injuries, and postsurgical repair [70,71,72].

To date, it is also widely used in aesthetic medicine and cosmetology in rejuvenating procedures in relation to its ability to promote fibroblast proliferation, epithelial cell proliferation, and angiogenesis through the release of several types of cytokines, growth factors, and interferons [73,74,75]. Moreover, PRP seems to have anti-inflammatory effects related to the suppression of cyclooxygenase and to the production of prostaglandins [73,74,75].

PRP is produced with minimal manipulation and is considered safe and natural because it comes from an autologous source, with few risks of infection and without immune reactivity [76]. When injected, PRP is able to form a low viscosity gel that allows platelets to locally deliver and maintain high concentrations of growth factors.

PRP preparations contain about 1100 different proteins, including growth factors, and 1,000,000 platelets/μL, that can boost physiological homeostatic processes and also create further scaffolds for the reparatory and remodelling processes [77].

In vitro and animal studies have shown that platelet preparations can induce the proliferation of human mesenchymal stem cells and the synthesis of type I collagen, improving the wound healing process through the secretion of a platelet-derived growth factor (PDGF), vascular endothelial growth factor (VEGF), transforming growth factor (TGF), and epidermal growth factor (EGF) [78].

In aesthetic practices, PRP injections are currently being performed for skin rejuvenation, mainly for the treatment of nasolabial and crow’s feet wrinkles [79].

The PRP-induced scaffolds can increase the concentration of growth factors at the targeted site, guiding the collagen deposition [80]. An in vitro study showed that the platelets continue to synthesize and release growth factors for the first 7 days and allow the maturation of collagen fibres for about 10 weeks [80].

An RCT by Hu et al. [81] analysed the effects of injections of PRP in the mid-cheek region and nasolabial fold on one side of the face and of saline on the contralateral side and reported a significant between-group difference in skin texture (*p*-value = 0.004) [81].

Another RCT by Shin et al. [82] was conducted to assess the effects of PRP combined with fractional laser therapy for the treatment of ageing skin. The group who underwent PRP and fractional laser therapy reported increased skin elasticity and a decreased erythema index compared to the group who underwent fractional laser therapy alone. Moreover, PRP increased the number of fibroblasts and amount of collagen assessed after the histologic analysis of skin biopsies [82].

The growth factors contained in PRP preparations seem to counteract the effects of dihydrotestosterone that are responsible for androgenic alopecia. Thus, several studies have been conducted to investigate the effects of PRP injections on hair density and hair shaft thickness in androgenic alopecia patients [83,84,85].

An RCT conducted by Balasundaram et al. [86] assessed the efficacy of PRP preparation (administered for 3 months) compared to topical minoxidil (administered for 6 months) in a sample of 64 males affected by androgenic alopecia and showed a significant increase in hair count and density, without between-group differences [86].

A double-blind RCT by Wei et al. [87] assessed the efficacy of PRP combined with topical 5% minoxidil therapy (administered for 3 months) in a sample of 30 males and detected a significant increase in hair density and quantity, suggesting that the combined therapy was superior to monotherapy in terms of patient satisfaction and clinical efficacy [87].

It must, however, be kept in mind that PRP effectiveness can vary significantly due to factors such as differences in blood harvesting techniques and variations in commercial PRP systems [88]. Indeed, these systems utilize various anticoagulants, volumes, centrifugation speeds, and processing steps, resulting in diverse PRP products with distinct characteristics. Key variables influencing PRP efficacy include the volume of PRP, platelet and leukocyte concentrations, the dose of injected platelets, and the capacity of the device to recover platelets from blood. Additionally, the activation process and overall composition of PRP play crucial roles in its therapeutic potential. While international biological classifications aim to standardize PRP usage, their adoption in clinical practice remains limited due to cost and resource constraints [88].

In conclusion, it should be taken into account that the rejuvenation of facial skin may represent another potential application for PRP.

### 4.3. Hyaluronic acid Therapy

Hyaluronic acid (HA) is a non-sulphated glycosaminoglycan constituted by a repetition of polymeric disaccharides of D-glucuronic acid and N-acetyl-D-glucosamine linked via glycoside bonds in an alternating fashion of β-(1 → 4) and β-(1→ 3) [89,90].

HA possesses a remarkable ability to retain approximately 1000 times its weight in water. It is located at the periphery and interfaces of collagen and elastin fibres, playing a role in maintaining the proper configuration of collagen and elastin [91,92].

In aged skin, the connections with HA are notably weak or even non-existent. This deficiency can contribute to the disarray of collagen and elastin fibres, ultimately causing the appearance of fine lines, wrinkles, and nasolabial folds in the skin [93]. Ageing is a natural process that gradually leads to the weakening of biological functions and the capacity to adapt to metabolic stresses, ultimately leading to senescence or old age. As one ages, tendons, blood vessels, and skin experience a reduction in elasticity. This occurs because cross-links form between or within collagen molecules, altering the structural proteins and creating impediments to normal cellular functioning. Among various symptoms, the loss of facial volume stands out as a characteristic feature of the ageing process.

Several studies have shown that the subcutaneous injection of HA enhances facial volume, playing a significant role in facial rejuvenation [94,95,96].

The results are evaluated through an assessment of skin elasticity, moisture, and turgor, revealing that natural HA has beneficial effects in improving both skin elasticity and turgor [97]. Skin health is significantly influenced by factors such as skin hydration. Another appealing aspect linked to the intradermal injection of HA filler is the stimulation of collagen production [98].

Collagen, in fact, is the major structural protein of the dermal extracellular matrix, conditioning skin tone, texture, and appearance.

Beyond aesthetics, HA serves various purposes in the rehabilitation process. In the treatment of edentulism, it can be complemented with microfocused ultrasound, radiofrequency, botulinum toxin, and prosthetics [99]. The use of HA fillers in the peri-oral area, including the lips, nasolabial and labiomandibular folds, and labiomental crease, enables physicians to provide a more comprehensive and natural treatment for edentulous patients. This is particularly beneficial when prostheses alone are insufficient to address advanced bone loss. The applications of HA fillers in these cases should be considered as part of oral rehabilitation treatment, restoring the patient’s natural appearance that might have otherwise been lost. In this scenario, oral rehabilitation should be taken into consideration also in patients affected by neurological diseases [100,101]. Furthermore, another area of application of HA filler as rehabilitative aesthetic medicine is represented by the treatment of acquired facial lipoatrophy [102], a condition consisting of loss of facial volume and harmony related to several conditions, such as highly active antiretroviral therapy (HAART) for HIV infection and connective tissue diseases such as lupus erythematosus and localized scleroderma among others [102].

Moreover, hyaluronic acid is utilized in breast reconstructive surgery, as demonstrated by Panettiere et al., who administered injections to 70 patients following nipple reconstruction to enhance nipple projection [103].

In conclusion, with promising cosmetic and nutricosmetic efficacy, an acceptable safety profile, biocompatibility, and enhanced patient compliance, formulations based on hyaluronic acid are justified in different settings, especially for treating various skin defects and as an anti-ageing modality [104].

### 4.4. Ozone Therapy and Carboxytherapy

Ozone was utilized in medicine in an empirical and imprecise manner for approximately 200 years. Over the past decade, advancements in technology have allowed the development of medical ozone generators capable of accurately determining ozone concentrations in real time and providing clarity on the chemical actions of ozone [105,106]. Ozone is an unstable molecule composed of three oxygen atoms, which can disintegrate into O_2_ and a single oxygen atom. This singular oxygen atom acts as a potent oxidant and anti-inflammatory agent [105,106].

Since ozone can inactivate bacteria, viruses, and spores [107] it can be effective in treating infected wounds [108]. In animal experimental models, ozone therapy has been confirmed to have beneficial effects when used as an adjunct to standard antibiotic treatment [109]. It has found applications in aesthetic medicine as well due to its anti-microbial, anti-inflammatory properties and its capability to remove pollutants, as presented by Lacerda et al. [110], who obtained an improvement in skin texture and a reduction of skin ageing signs in a 73-year-old female after six sessions of topical ozone therapy in its gas form. The dose was low (5 mcg/mL) during the first two sessions, medium (10 mcg/mL) during the third and the fourth sessions, and high (15 mcg/mL) during the fifth and sixth sessions. Each session lasted about 40 min and was performed once a week for a total of six weeks [110]. In spite of this knowledge, multicentric clinical trials are still needed to ensure ozone therapy reliability and practicability.

Carboxytherapy is a medical treatment that consists of exploiting carbon dioxide (CO_2_) to obtain therapeutic effects. It was initially used in French spas in the 1930s. Recently, it has attracted clinical interest due to its application in aesthetic medicine [111].

It is thought to lead to skin regeneration by increasing the blood supply, dilating capillaries and pre-capillary vessels, increasing oxygen levels through the Bohr effect, increasing red blood cell deformability, and having a possible antiseptic effect due to the decrease in local pH [112].

Devices are required that can provide variable pressure with stable but adjustable flow, ensuring delivery of sterile pre-warmed gas [113].

For the face and neck, it is preferable to use needles with a length of 4 to 6 mm and a gauge of 30. For deeper treatments, such as cellulite treatment on thighs and buttocks, needles with a length of 13 mm are recommended [113].

Aksenenko et al. [114] applied injective carboxytherapy as rehabilitation treatment in patients who had developed oedema and neuropathy in the facial region after receiving radiofrequency to treat skin ageing signs, revealing an ultrasonographic reduction of skin oedema and an improvement in facial neuropathy, referred to by patients as a decrement in facial pain [114]. It is a versatile, cost-effective, and “natural” treatment, but results can be unpredictable and so caution must be used.

## 5. Physical Therapies in Aesthetic Rehabilitation

**Figure 1:**
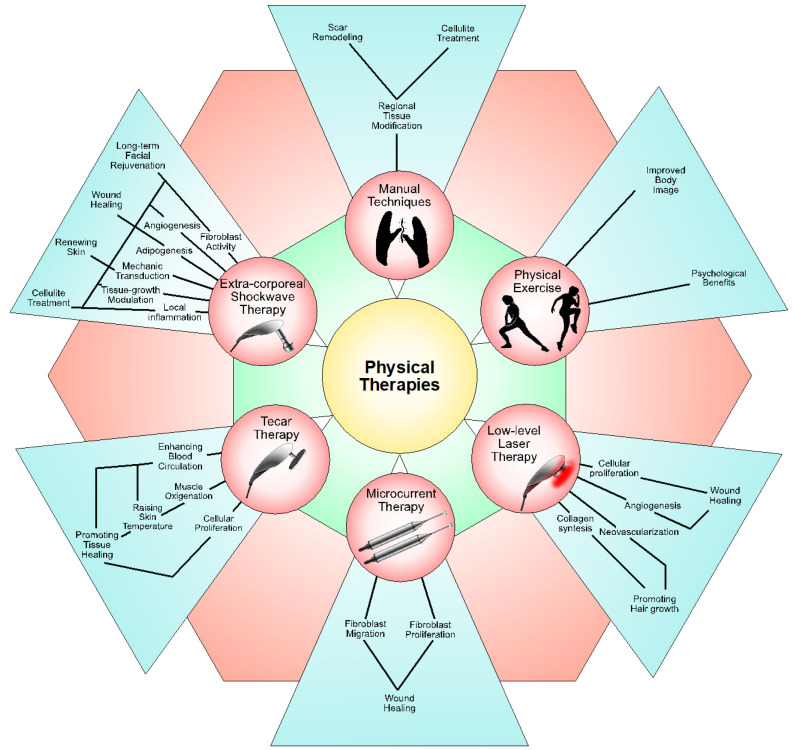
A summary of the different physical therapies used in the rehabilitation field and their biological impact.

Physical therapies play a pivotal role in addressing both functional impairments and aesthetic concerns. From techniques targeting subcutaneous adipose tissue to specialized interventions aimed at wound and scar management, physical therapies offer benefits that extend beyond pain relief to include improvements in aesthetics. Figure 1 shows the different physical therapies utilized in the rehabilitation field and their corresponding biological effects.

### 5.1. Extracorporeal Shock Wave Therapy (ESWT)

Extracorporeal shock wave therapy (ESWT) was initially developed for lithotripsy in the 1970s and later adapted and used for orthopaedic disorders [115]. It acts by providing a mechanical stimulus that promotes healing via mechanotransduction, leading to angiogenesis, tissue regeneration, and bone remodelling at cellular and molecular levels [116]. These mechanisms contribute to pain relief, reduction of inflammatory mediators, absorption of calcifications, and chondroprotective effects, making it a valuable non-invasive treatment modality, and, ultimately, for use in regenerative medicine [116]. In fact, ESWT triggers cellular growth and protein synthesis, favouring the regeneration of injured tissues [116]. Exploiting this concept, it began to be implemented in aesthetic treatments, targeting localized fat, cellulite, and loose skin [115]. In simple terms, the method for addressing aesthetic issues works by encouraging fibroblast activity through mechanical signals, which in turn prompts the production of growth factors crucial for renewing skin tissue [115]. However, the precise mechanism of ESWT remains unclear.

With these premises, ESWT could be used as an enhancer of functional and aesthetic aspects of the skin. While ESWT has proved to be effective in promoting cutaneous microcirculation on animal models, both immediately after treatment and even in remote sites [117,118], a 2022 study by Modena et al. showed the effect of ESWT on integumentary tissue of individuals with grade II obesity. The study reported a significant increase in epidermal expression of inflammatory markers (*p* < 0.0001) compared to controls, along with positive expression for angiogenesis markers (*p* < 0.0001). These findings suggest that ESWT can stimulate a local inflammatory process, modulating crucial growth factors and showing promise as a treatment for skin conditions associated with weight changes [115].

In this context, cellulite is defined as a cosmetic condition characterized by the presence of a dimpled or puckered appearance of the skin, primarily affecting post-pubertal women in areas like the thighs, buttocks, and abdomen [119]. The aetiology, though not completely understood, is often considered multifactorial, involving gender, anatomical and hormonal factors, genetic predisposition, lifestyle, and deficiencies in lymphatic drainage and microvasculature [119]. Moreover, a study on histological aspects of cellulite proposed that cellulite is a degenerative condition, as different histological aspects are concomitantly present in the same area [119]. A 2015 meta-analysis reviewed eleven clinical studies on ESWT for cellulite treatment. Increasing evidence suggests that both radial and focused ESWT, either alone or in combination, ranging from six to eight treatments administered once or twice weekly, can improve cellulite appearance [120].

Moreover, a more recent 2017 systematic review concluded that ESWT induces significant effects on biological tissues, resulting in the restructuring of skin properties and subcutaneous tissue. This, in turn, leads to clinical improvements in cellulite and localized fat [121]. Furthermore, ESWT was also proposed as an adjunct to cryolipolysis to achieve non-invasive body contouring, with positive results [122]. A 2021 RCT compared the effectiveness of ESWT and manual lymphatic drainage in reducing cellulite post-liposuction. Thirty females with grade 3 cellulite were divided into two groups. Results showed a more significant reduction in cellulite grade and subcutaneous fat thickness in the ESWT group compared to the manual lymphatic drainage group, indicating ESWT’s superiority in cellulite reduction after liposuction [123].

The regenerative properties of ESWT have also been exploited in the context of restoring volume, a key aspect of facial rejuvenation. One technique used is autologous fat grafting. A study by Priglinger et al. demonstrated that ESWT applied to the adipose tissue harvest site could enhance cell fitness, adipogenesis, and angiogenesis within fat grafts, potentially leading to successful and long-term facial rejuvenation [124].

Wound healing is a complex physiological process involving numerous cells and factors, critical for injury repair and wound closure. Failure to address influencing factors may lead to impaired wound healing and the development of chronic wounds, significantly impacting the patient’s QoL and placing a substantial burden on healthcare systems [125]. ESWT has shown promising results in wound healing, ranging from ulcers to burn injuries [126,127]. In animal models, ESWT improved skin regeneration of deep partial-thickness burns [127]; in human studies, it increased perfusion of the burnt area [128]. These regenerative properties could make ESWT a suitable and cost effective treatment alternative for burns [127]. Moreover, clinical studies and animal experiments have demonstrated positive outcomes including accelerated wound closure and increased survival time of skin flaps [129,130,131]. Likewise, ESWT could be considered effective and safe in skin wound treatment. This is proved by an initially accelerated rate of wound closure in the treatment groups, which later equals the closure rate observed in the control group. Additionally, a notable enhancement in microcirculation and perfusion during the healing process was observed, implying an ESWT-induced improvement in nutrient and oxygen supply to the wound tissue [125].

In this context, wound treatment is considered an important part of an after-surgery or trauma-healing rehabilitation programme, focusing on the overall wellbeing and QoL experienced by the affected individuals [132]. In some cases, a hypertrophic scar develops due to an abnormal wound healing process, characterized by an overproduction of dermal collagen. This can result in various challenges for individuals, including tenderness in the scar area, itching, and restrictions in the range of motion. These factors collectively have an adverse effect on QoL [132]. ESWT could be utilized for hypertrophic scar treatment with promising results, including functional (pain, itching, and pliability) and aesthetic (pigmentation) results [132].

Lastly, ESWT could also be implemented to treat side effects of common aesthetic procedures. A 2021 case study reported how two patients were successfully treated for delayed-onset nodules, a potential complication associated with injectable HA fillers, with up to four sessions of ESWT [133]. This complication can cause concern for both patients and clinicians. ESWT allowed for maintenance of the aesthetic effects of the HA filler, resulting in patient satisfaction [133]. Furthermore, capsular fibrosis is a common long-term complication attributed to inflammatory reactions and extracellular matrix formation due to silicone device insertion [134]. ESWT was used with positive results to treat capsular fibrosis after augmentation of the female breast with silicone implants, working as a non-invasive and well-tolerated treatment option for fibrotic tissue softening and pain reduction [135]. An animal model study determined that multiple ESWT applications resulted in a significantly thinner capsule at 100 days post-insertion, surpassing the effect of a single application. This active degradation of fibrous tissue was associated with alterations in pro- and anti-fibrotic proteins, suggesting the potential of ESWT to influence inflammation and fibrotic processes after silicone implantation [134].

Most notably, all interventional studies reported a high tolerability of the procedure, making it a feasible and safe option [115,122,132,133,135].

### 5.2. Laser Therapy

Low-level laser therapy (LLLT), also known as Photobiomodulation (PBM), utilizes low-intensity light for its photochemical effects. This technique induces biochemical changes in cells by activating cellular photoreceptors, thereby fostering the proliferation of various cell types, including fibroblasts, keratinocytes, endothelial cells, and lymphocytes [136,137]. This process involves photo-stimulation of mitochondria, triggering signalling pathways and enhancing transcription factors that lead to increased growth factors [136]. Demonstrating its versatility, LLLT supports neovascularization, angiogenesis, and collagen synthesis [136], and so finds applications across various medical fields. These include regenerative medicine for wound and ulcer healing, physiotherapy for chronic pain reduction, orthopaedics in bone healing, cardiology for preventing restenosis after percutaneous coronary intervention, dentistry for expedited implant healing, and aesthetic medicine for scar appearance improvement [138]. Positive results have also been recorded in repigmentation of vitiligo-affected skin [139]. Notably, the efficacy of LLLT varies with wavelength, with shorter wavelengths effective for deeper tissue injuries and longer wavelengths suitable for superficial traumas [140]. However, handling lasers requires caution and experience, since side effects are common. These include burns, infections, scarring, erythema and contact dermatitis, worsening of the original condition, dyspigmentation, and eye injuries [141].

As emphasized earlier, wound management is pivotal in rehabilitation medicine, and LLLT serves both functional and aesthetic purposes in this context. Angiogenesis in burn wounds, assessed using an in vitro model, demonstrated PBM’s efficacy in promoting angiogenesis on day 4 (*p* = 0.005), with a subsequent non-significant trend toward higher angiogenesis [142]. On the other hand, in an animal study comparing adipose-derived stem cells (ADST), LLLT groups and their combinations all exhibited significant improvements in re-epithelialization and overall healing processes, with superior results in the combined therapy group, showing reduced inflammatory phases, increased angiogenesis, decreased oedema, enhanced collagen deposition, and improved extracellular matrix organization [143]. This combination has also shown effectiveness in in vitro studies of PBM-treated ADST for wound healing [144], and LLLT with human amniotic membrane mesenchymal stem cells improved chronic wound conditions in an animal model [145]. PBM was found to be effective in improving diabetic foot ulcers and non-healing ulcers in humans, as an adjunct to routine ulcer care [146].

A randomized clinical trial on burn patients undergoing skin graft surgery demonstrated that treating the surgical wound with LLLT was a safe and effective method, improving graft survival and the wound healing process, and reducing scar dehiscence risk [147]. These results underscore LLLT’s efficacy as an adjunctive treatment.

An animal study employing PBM to explore hair regeneration in injured skin significantly increased hair density compared to the control group, indicating its potential role in promoting hair regeneration [148]. In this context, LLLT could address hair loss, with a positive impact on mental and psychological health, including chemotherapy-induced hair loss [149,150]. LLLT yields interesting results, especially in terms of decreased hair loss and increased hair growth, though possibly causing mild side effects such as tenderness, paraesthesia, and mild urticaria of the scalp. More research is warranted [151].

While non-invasive, cost-effective, and versatile LLLT devices show promise in skin pathologies, there is a pressing need within the healthcare community to establish optimal clinical protocols through well-designed and rigorous research studies [150,152].

### 5.3. Microcurrent Therapy

Microcurrent therapy (MCT) involves the application of minimal electric currents, measuring less than 1 mA, applied to cutaneous surfaces without causing muscle contractions or perceivable sensations [153]. It has demonstrated the ability to enhance the migration, proliferation, and differentiation of human dermal fibroblasts. Additionally, electrical stimulation has been observed to stimulate collagen synthesis, fibroblast proliferation, and migration [154]. Animal studies have shown its efficacy in promoting muscle and tendon regeneration, either alone [155,156] or as an adjunctive therapy [157,158].

Positive results were obtained with MCT by expediting the process of wound healing [154]. A 2022 study showed the potential of MCT in facilitating the healing of pressure injuries [154].

A preliminary study was conducted to assess the efficacy of MCT in the management of treatment sequelae in individuals who have undergone treatment for head and neck cancer, specifically progressive fibrosis observed in the soft tissues of the radiotherapy-treated area. These could result in a restricted range of motion and/or pain during movement [159]. Notably, MCT yielded positive results in addressing these issues [159].

### 5.4. Tecar Therapy

Tecar (transfer energy capacitive and resistive) therapy has recently been the subject of studies investigating its effectiveness in the rehabilitation of various musculoskeletal conditions [160,161]. This technology utilizes high-frequency electromagnetic waves ranging from 0.3 to 1.2 MHz, aimed at reducing spasms and contractions induced by muscle activity, enhancing blood circulation in the treated tissue, and promoting muscle oxygenation by increasing haemoglobin [160]. As a result, Tecar therapy could increase tissue healing and pain alleviation [160]. In a cadaveric study, low-power applications generated a minimal thermal effect but a significant current flow, while high-power capacitive and resistive applications demonstrated a substantial increase in superficial temperature, affecting superficial, middle, and deep temperatures, along with greater current flow [161]. This mechanism was proposed to be the means of potentially accelerating muscle recovery, enhancing cell proliferation in acute injuries, and improving tissue temperature and viscoelasticity in chronic conditions [161].

Confirming these findings, both skin microcirculation and intramuscular blood flow were found to be affected by Tecar therapy [162]. Moreover, the resistive mode induced significative changes in intramuscular blood flow and skin temperature, providing valuable insights into the therapy’s potential to affect blood flow, a crucial mechanism for promoting tissue healing processes [162].

Additionally, Tecar therapy action was assessed on biomarkers of skin fibrosis using human myofibroblast cultures. Results indicated that it decreased the expression of extracellular matrix proteins, altered metalloproteinase 9 expression, and reduced nuclear factor kappa-light-chain-enhancer of activated B cells activation compared to controls, suggesting its potential efficacy in treating fibrotic conditions [163].

Interestingly, a synergistic approach involving therapeutic modalities such as Tecar therapy and exercise may offer effective solutions in reducing adipose tissue in various clinical settings [164]. In fact, radiofrequency devices, such as Tecar [165], could utilize high-frequency electromagnetic energy to efficiently heat adipose tissue, thereby boosting local cell metabolism and increasing lipolysis [164]. The combined intervention of aerobic exercise with radiofrequency demonstrated a statistically significant decrease in waist circumference, abdominal subcutaneous adipose thickness, and horizontal abdominal folds compared to a placebo [164]. This suggests that adopting a combined approach involving exercise alongside Tecar therapy could enhance the efficiency of lipid removal during targeted interventions [164].

Moreover, Tecar therapy was found to be an effective and potentially early intervention for lower limb oedema in morbidly obese patients, improving patients’ function and reducing pain [166].

In conclusion, these studies collectively provide valuable insights into the potential benefits of Tecar therapy in aesthetic rehabilitation. The multidisciplinary approach demonstrated in the studies highlights the versatility of this treatment in addressing diverse aspects of rehabilitation and promoting positive aesthetic outcomes.

### 5.5. Physical Exercises

Physical exercise plays an important role in enhancing overall quality of life. Nowadays, sedentary lifestyles represent a rising issue, negatively impacting physical, mental, and social wellbeing [167]. Increasing physical activity promotes self-awareness and improves body image [167,168].

The intersection of physical exercise as a rehabilitative intervention and its impact on aesthetic concerns is highlighted in several studies. In one study involving women with obesity, regular physical activity over a three-month period led to a significant improvement in body shape perception and lowered body shape concerns [169]. Similarly, another study examined the effects of aerobic physical exercise on body image among women with polycystic ovary syndrome [170]. While no significant changes were observed in the perceptual dimension of body image, aerobic exercise improved indices related to anxiety, depression, and sexual function, suggesting a positive impact on cognitive–affective aspects of body image [170]. Furthermore, the effects of interdisciplinary therapy were evaluated on neuroendocrine control, inflammation, and psychological factors in obese women [171]. The findings indicated that the therapy, which included physical exercise among other interventions, not only resulted in physical benefits such as weight loss and improved body composition but also provided psychological benefits, including enhanced body image perception and reduced depression and anxiety scores [171].

While traditionally viewed as a means to address physical health and functional concerns, physical activity interventions hold significant promise in addressing aesthetic and psychological aspects of wellbeing. By promoting positive body image perception, reducing body shape concerns, and improving psychological parameters such as anxiety and depression, physical exercise emerges as a multifaceted tool for comprehensive rehabilitation.

### 5.6. Manual Techniques

The overlap between manual techniques as rehabilitative interventions and aesthetic interests is a multifaceted phenomenon that bridges the gap between therapeutic goals and aesthetic outcomes. Various studies underscore the effectiveness of manual therapy in addressing both physical discomfort and aesthetic concerns. For instance, manual subcutaneous adipose tissue therapy not only reduces pain but also enhances the structure of subcutaneous adipose tissue, showing the potential for aesthetic improvements alongside pain relief [172]. Similarly, manual techniques on fat mass in women with cellulite revealed a significant reduction in regional fat thickness, thus highlighting the aesthetic benefits of manual therapy techniques [173]. Furthermore, soft tissue mobilization on scar tissue demonstrated improvements in scar structure, elasticity, and pliability, suggesting a potential enhancement in aesthetic appearance following manual therapy interventions [174,175,176]. These findings collectively emphasize the dual role of manual therapy techniques in addressing rehabilitative needs while simultaneously resulting in aesthetic improvements, thereby once again highlighting the interconnection between rehabilitation and aesthetics.

## 6. Conclusions

Altogether, this review provides a throughout examination of the wide range of rehabilitative techniques and interventions commonly employed in rehabilitation settings, with a primary emphasis on their capacity to enhance aesthetics, quality of life, and psychosocial wellbeing among individuals facing disabilities. From intricate reconstructive efforts post-trauma or surgery to the targeted application of injection therapies and physical modalities, each factor plays a pivotal role in fostering holistic rehabilitation outcomes.

The integration of aesthetic approaches within the field of rehabilitation underscores a synergistic approach to patient-centric care. These interventions, addressing functional impairments and quality of life, aim to enhance both physical and psychosocial wellbeing among individuals with disabilities. Therefore, aesthetic considerations are strongly recommended to be integrated into rehabilitation programmes.

## Figures and Tables

**Table 1:** Spider tool search strategy.

S	PI	D	E	R
Sample	Phenomenon of Interest	Design	Evaluation	Research Type
Patient undergoing Aesthetic Rehabilitation Treatments	Physical Therapies	Any	Functional outcomes and Quality of Life	Qualitative
	“Aesthetic Rehabilitation” “Physical Therapies” “Botulinum Toxin” “Laser Therapy” “Tecar Therapy” “ESWL”		“Function” “Quality of Life”	

## Data Availability

Data sharing is not applicable to this article.
